# MIIP inhibits the growth of prostate cancer via interaction with PP1α and negative modulation of AKT signaling

**DOI:** 10.1186/s12964-019-0355-1

**Published:** 2019-05-15

**Authors:** Guang Yan, Yi Ru, Fengqi Yan, Xin Xiong, Wei Hu, Tao Pan, Jianming Sun, Chi Zhang, Qinghao Wang, Xia Li

**Affiliations:** 1 State Key Laboratory of Cancer Biology, Department of Biochemistry and Molecular Biology, The Fourth Military Medical University, Xi’an, 710032 Shaanxi China; 2 Andrology Department, Shanghai Seventh People’s Hospital, Shanghai, 200137 China; 3 Department of Urology, Tangdu Hospital, The Fourth Military Medical University, Xi’an, 710038 Shaanxi China; 4 Rehabilitation Department, Gongli Hospital of Shanghai Pudong New Area, Shanghai, 200137 China

**Keywords:** MIIP, Proliferation, Prostate cancer, PP1α, PI3K-AKT-mTOR

## Abstract

**Background:**

Over-activation of phosphatidylinositol 3-kinase (PI3K)-AKT-mammalian target of rapamycin (mTOR) signaling pathway is one of important mechanisms to promote castration resistant prostate cancer, the final stage of prostate cancer (PCa). Dysregulation of PP1-meditaed AKT dephosphorylation might contribute to such an event but is not fully understood. As a newly identified tumor suppressor, MIIP exerts its role in various types of cancer but has not been investigated in PCa.

**Results:**

We first demonstrated that overexpression of migration and invasion inhibitory protein (MIIP) in human PCa cell lines suppresses their growth while knockdown of MIIP does the opposite in vitro. Although MIIP has no effect on the expression of AR and its target genes or the nuclear translocation of AR in AR-positive PCa cells, MIIP overexpression significantly inhibits activation of AKT-mTOR pathway in both AR- positive and negative PCa cells whereas knockdown of MIIP enhances AKT-mTOR signaling. Using Western blot, immunofluorescence co-localization and co-immunoprecipitation analysis, we found that MIIP interacts with PP1α via its C-terminal part but does not affect its protein level. Importantly, silence of PP1α reversed the inhibitory effect of MIIP on AKT phosphorylation and cell growth in PCa cell lines, while MIIP∆C, which is incapable of interacting with PP1α, loses MIIP’s effect, suggesting that MIIP exerts its roles via interaction with PP1α. Further, MIIP overexpression inhibits the growth of both AR- positive and negative PCa xenograft in nude mice. Finally, immunohistochemical staining of PCa tissue microarray showed that MIIP expression level is downregulated in PCa and negatively correlated with Gleason score of PCa.

**Conclusion:**

We discovered that MIIP is a novel suppressor of oncogenic AKT-mTOR signaling in PCa by facilitating PP1-meditaed AKT dephosphorylation. Our study further emphasized the tumor suppressive role of MIIP and illustrated a novel mechanism.

**Electronic supplementary material:**

The online version of this article (10.1186/s12964-019-0355-1) contains supplementary material, which is available to authorized users.

## Background

Prostate cancer (PCa) is the second most common malignancy worldwide in men and the fifth leading cause of cancer death [1]. Androgen receptor (AR), as a nuclear receptor, plays an essential role in the initiation and progression of PCa [2]. Upon ligand binding, AR translocates to the nucleus and activates a set of AR target genes. As such, androgen-deprivation therapy (ADT) has been used as a first-line treatment for metastatic PCa and achieved significant clinical response [3]. However, nearly all patients eventually become resistant to ADT and progress to castration resistant prostate cancer (CRPC), which is the final stage of PCa [4]. Studies on the molecular mechanisms of CRPC have revealed that reactivation of AR signaling axis is a predominant reason, which includes AR gene amplification/overexpression, AR mutations conferring ligand promiscuity, variant AR isoforms that are ligand-independent, and intratumoral androgen biosynthesis [5, 6]. Therefore the novel inhibitors targeting AR or androgen synthesis have been applied and resulted in survival benefit to some extent in patients with CRPC [4, 7, 8]. However, advanced PCa remains uniformly fatal, highlighting the urgent need for additional exploration of the mechanism and therapy besides the AR signaling axis.

The phosphatidylinositol 3-kinase (PI3K)-AKT-mammalian target of rapamycin (mTOR) signaling, as a key oncogenic pathway in various cancers, is clearly emerging as another important mechanism to promote CRPC [9]. In fact, activation of PI3K-AKT-mTOR pathway occurs in 42% of primary prostate tumors and 100% of metastatic tumors [10]. Thus targeting PI3K-AKT-mTOR is considered a promising approach to treat PCa, particularly CRPC [11, 12]. In addition to genomic and transcriptional alteration of its components, PI3K-AKT-mTOR signaling is frequently activated due to genetic loss or inactivation of several phosphatases that antagonize this pathway, including phosphatase and tensin homolog gene (PTEN), INPP4B, protein phosphatase 2A(PP2A) and the PH-domain leucine-rich-repeat-containing protein phosphatases (PHLPP1/2) [11]. Among them PP2A and PHLPP directly dephosphorylate serine/threonine kinase AKT, which is activated via phosphorylation at Thr308 by PDK1 and at Ser473 by mTORC2 [11, 13]. Recently, another protein phosphatase PP1 has also been reported to interact with and dephosphorylate AKT [14, 15]. Moreover, PP1-dependent dephosphorylation and inactivation of AKT can be hijacked by some oncoproteins and targeted by anticancer agents [14–16]. Although it was sparsely reported that inhibition of PP1-mediated AKT dephosphorylation contributes to AKT activation in PCa [14], the exact role and regulation of such an event is not fully understood.

Migration and invasion inhibitory protein (MIIP), also known as invasion inhibitory protein 45 (IIp45), is recently identified as a tumor suppressor. *MIIP* gene is located at chromosome 1p36.22, which is a frequently deleted region in numerous cancers, and encode a cytosolic protein with molecular weight of 45 kDa [17]. The expression level of MIIP is decreased in many types of cancer, including glioma, lung cancer, colon cancer, endometrial carcinoma and pancreatic cancer, and correlates with advanced clinical stage and shorter survival time of the patients [17–22]. The amounting evidences has established that MIIP inhibits cell migration and invasion by associating with and regulating IGFBP-2 [20], histone deacetylase 6 (HDAC6) [23], or PAK1 [18]. MIIP is also able to attenuate mitotic transition and cell proliferation by interacting with Cdc20 and blockage of Cyclin B1 degradation [24]. In addition, MIIP can interact with topoisomerase II (Topo II) and maintain its activity and chromosomal stability [25]. These findings highly suggest that MIIP exerts tumor-suppressive role in a multifaceted way via different molecular mechanism. However, its biological function and mechanism have not been elucidated in PCa.

In this study, we attempted to explore the role and the mechanism of MIIP in PCa. We showed here that overexpression of MIIP inhibit prostate cancer cell growth both in vitro and in nude mice. Mechanistically, MIIP does not affect AR signaling but attenuates AKT-mTOR axis by interacting with Serine/Threonine Protein phosphatases PP1α. Importantly, the immunohistochemical staining result of PCa tissue microarray showed that the expression level of MIIP is negatively associated with Gleason score.

## Methods

### Patients’ samples

Tissue microarrays (TMAs) containing 8 cases normal adults samples and 73 cases patients samples [170 points: 30 normal (16 normal prostate tissue plus14 adjacent normal prostate tissue), 23 Gleason score 3, 71 Gleason score 4, and 46 Gleason score 5] were commercially obtained from Xi’an Alenabio Technology Co., LTD., and the experiments were approved by Research Ethics Committee. Another independent TMAs containing 34 PCa patients samples (68 points: 22 Gleason score 3, 26 Gleason score 4, and 20 Gleason score 5) were obtained from the Department of Pathology of Xijing Hospital with informed consent of the patients, and approval from the Clinical Research Ethics Committee of Xijing Hospital, the Fourth Military Medical University.

### Cell lines, antibodies and reagents

Human PCa cell lines LNCaP, C4–2, 22Rv1 and PC3 were purchased from Chinese Academy of Sciences Cell Bank (Shanghai, China) and maintained in RPMI-1640 (Invitrogen Grand Island, NY, USA). Human embryonic kidney cell HEK-293 T and human cervical cancer cell line HeLa were purchased from American Type Culture Collection (Manassas, VA, USA), and maintained in DMEM (Invitrogen, Grand Island, NY). All mediums were supplemented with 10% fetal bovine serum (FBS, Gibco, Australia), 100 U/mL penicillin (Sigma-Aldrich, St. Louis, MO, USA) and 50 μg/mL streptomycin (Sigma-Aldrich, St. Louis, MO, USA). All cell lines were cultured in incubators with humidified atmosphere of 5% CO_2_ and 95% air at 37 °C. Anti-MIIP and anti-FLAG antibody were from Sigma-Aldrich (St. Louis, MO, USA). Anti-PP1α was from Sata Cruz Biotechnology (Dallas, Texas, USA). Anti-HA was from Abcam (Cambridge, MA,USA). Anti-PI3K(p110), anti-AKT, anti-p-AKT (Ser473), anti-mTOR, anti-p-mTOR (Ser2448), anti-p-mTOR (Ser2448), anti-AR, anti-GAPDH and anti-α-tubulin antibodies were from Cell Signaling Technology (Beverly, MA, USA). Dihydrotestosterone, Enzalutamide and BKM120 were commercially purchased (MedChemExpress, Princeton, NJ).

### Vector construction, cell transfection and lentivirus packaging

The *MIIP* CDS was amplified by PCR using the primers and cloned into pLEX-HA-MCS vector (Thermo Scientfic, Waltham, MA) with restriction enzymes *Spe* I and *Xho* I (New England Biolabs, Ipswich, MA). pFLAG-CMV4-MIIP∆C and pFLAG-CMV4-MIIP∆N were generated by amplifying the fragments encoding MIIP N-terminal 1–219 residues and C-terminal 220–388 residues using PCR from pLEX-HA-MIIP and inserting the fragments into the *EcoR* I/*EcoR* V site of pFLAG-CMV4, respectively. pEGFP-N3-MIIP was constructed by subcloning MIIP from pLEX-HA-MIIP into *Nhe* I/*Hind* III site of pEGFP-N3. pmCherry-C1-PP1α was generated by PCR and cloned into *EcoR* I/*BamH* I site of pmCherry-C1 vector. The primers used for the construction of the above vectors were listed in Additional file 1: Table S1. For lentiviral packaging, we used packaging system (1 μg pLEX-MCS, 0.2 μg VSVG and 1 μg △8.9) to co-transfect HEK-293 T with lipofectamine 2000 (Thermo Scientfic, Waltham, MA). The stable cell lines LNCaP-MIIP, C4–2-MIIP, and PC3-MIIP were obtained by lentivirus infection and puromycin (1 μg/mL) selection. Transient transfection of PCa cell lines were conducted with lipofectamine 2000 according to the manufacturer’s instruction.

### RNA interference

Chemically synthesized siRNA duplexes were obtained from GenePharma (Shanghai, China). Negative control siRNA sequence: 5′-UUCUCCGAACGUGUCACGUTT-3′; siPP1α#1: 5′-CUGCUGGCCUAUAGAUCATT -3′, siPP1α#2: 5′-GACGCUACAACAUCAAACUTT -3′, siMIIP#1: 5′-CCAAACCGGAGGAGUGUAUTT -3′, siMIIP#2: 5′-GACCAUGAAUGCGUGUACUTT -3′. SiRNA transfection were conducted with lipofectamine 2000 according to the manufacturer’s instruction.

### Immunohistochemistry

Immunohistochemistry was performed according to standard procedures. The primary antibodies anti-MIIP (1:200) and anti-p-AKT (Ser473) (1:100), ABC Kit and DAB (Vector, USA) were used for staining. The results were obtained by digital slice scanner (3DHISTECH, Hungary). Protein expression was analyzed by immunoreactivity scored. The total score = cell score × color score. The cell score standard used the number of cells with positive staining (≤5%: 0, 6–25%: 1, 25–50%: 2, 51–75%: 3, ≥75%: 4). The color score standard used the staining intensity (colorless: 0, mild: 1, moderate: 2, strong: 3).

### Real-time RT-PCR analysis

Total RNA was prepared using TRIzol (Sigma, USA) and reversely transcribed into cDNA with the PrimeScript™ RT Reagent Kit (TakaRa, Dalian, China). Real-time quantitative PCR was performed using a CFX96™ Real-time system (Bio-Rad, USA) and SYBR Premix Ex Taq (TakaRa, Dalian, China) according to the manufacturer’s instruction. Raw data were normalized to the internal *GAPDH* and presented as relative expression level calculated by 2^△△^Ct method. All primers for qRT-PCR are described in Additional file 1: Table S2. All experiments were performed in triplicate.

### Western blot analysis

Samples from lysates of cultured cells were resolved on 10–12% SDS-PAGE gels, transferred to nitrocellulose membranes and probed with anti-MIIP (1:2000), anti-AR (1:2000), anti-PI3K (p110) (1:1000), anti-AKT (1:1000), anti-p-AKT (Ser473) (1:2000), anti-mTOR (1:1000), anti-p-mTOR (Ser2448) (1:1000), anti-p-mTOR (Ser2481) (1:1000), anti-PP1α (1:250), anti-HA (1:2000), anti-FLAG (1:2000), anti-α-tubulin (1:1000) and anti GAPDH (1:1000), followed by either anti-rabbit or anti-mouse IgG secondary antibodies conjugated to horseradish peroxidase at a dilution of 1:5000 (Proteintech Group, USA). The signal of the protein bands were detected using the ECL system (Bio-Rad, USA). The band intensity was quantified by using Image J software (NIH, USA), wherein the relative values (GAPDH or tubulin as internal control) of the first bands were designated as 1.

### Cell proliferation and colony formation

Cells were seeded at 2000 cells per 96-well in septuplet and cell proliferation was estimated using the CCK-8 kit (Dojindo Laboratories, Japan) according to the manufacturer’s instructions. Colony formation was measured two weeks after seeding 200 cells per well in 6-well plates. Cell colonies were fixed and then stained with Giemsa for 20 to 30 min. The number of colonies was directly reported, and the formation ratio was calculated according to the following formula: colony formation ratio (%) = (colony number / seeded cells number) × 100%. The data were presented as the mean ± SEM.

All the above experiments were repeated at least three times independently.

### Co-immunoprecipitation

HEK-293 T cells were transiently transfected with pLEX-HA-MIIP, pCMV4-FLAG-MIIP∆C, or pCMV4-FLAG-MIIP∆N, together with pmCherry-C1-PP1α. LNCaP-HA-MIIP was transfected with pmCherry-C1-PP1α. Fourty-eight hours later, cells were lysed on ice in lysis buffer (30 mM Tris, pH 7.5, 150 mM NaCl, 1% Triton X-100). Protein lysates containing 1 mg total protein were incubated with 2 μg anti-HA, anti-FLAG or 2 μg mouse IgG for 6 h at 4 °C, followed by incubation with protein A + G sepharose IP beads (Santa Cruz Biotechnology, CA, USA) overnight at 4 °C. IP beads were subsequently washed four times with lysis buffer and boiled in SDS sample buffer for 10 min. Samples were then separated by SDS-PAGE followed by immunoblot with anti-PP1α and anti-MIIP or anti-FLAG.

### Co-localization analysis and immunofluorescence staining

HeLa cells were plated on cover slips and transfected with pEGFP-N3-MIIP and pmCherry-C1-PP1α, and co-localization analysis were completed by confocal microscopy after culturing for 48 h. LNCaP-HA-MIIP cells were transfected with pmCherry-C1-PP1α and cultured for 48 h before staining. After removal of culture media, cells were washed with PBS, fixed in 4% PFA, permeabilized with 0.2% Triton X-100 for 10 min and then blocked with 2% BSA in PBS for 1 h. Anti-MIIP (1:200) and anti- PP1α (1:100) primary antibodies were diluted in the blocking solution and applied overnight at 4 °C. After PBS wash for three times, secondary antibodies 488 and Cy3 (GeneTex, San Antonio, Texas) were diluted (1:100) in blocking buffer and applied for 1 h at room temperature. After PBS wash for three times, nuclei were stained with DAPI (1:4000) for 10 min. Co-localization analysis were performed by confocal microscopy.

### In vivo xenograft experiment

The animal studies were approved by the Animal Ethics Committee of the Fourth Military Medical University. Six-week-old male nude mice were injected subcutaneously in the limb with 1 × 10^7^cells. Tumor growth was monitored by measuring tumor size using vernier calipers every 10 days for 40 days period, and tumor volume calculated using a standard formula: tumor volume (mm^3^) = width (mm^2^) × length (mm) × 0.5. Tumor weight was assessed after sacrificing the mice. Tumor cell proliferation were analyzed by Ki67 staining (Abcam, Cambridge, MA). The data were presented as the mean ± SEM.

### Statistics and data analyses

Data are expressed as the mean ± SEM, and statistical evaluation was performed using the Student’s t-test for independent groups. Values of *p* < 0.05 were considered statistically significant.

## Results

### MIIP inhibits the proliferation of PCa cell lines

**Fig. 1:**
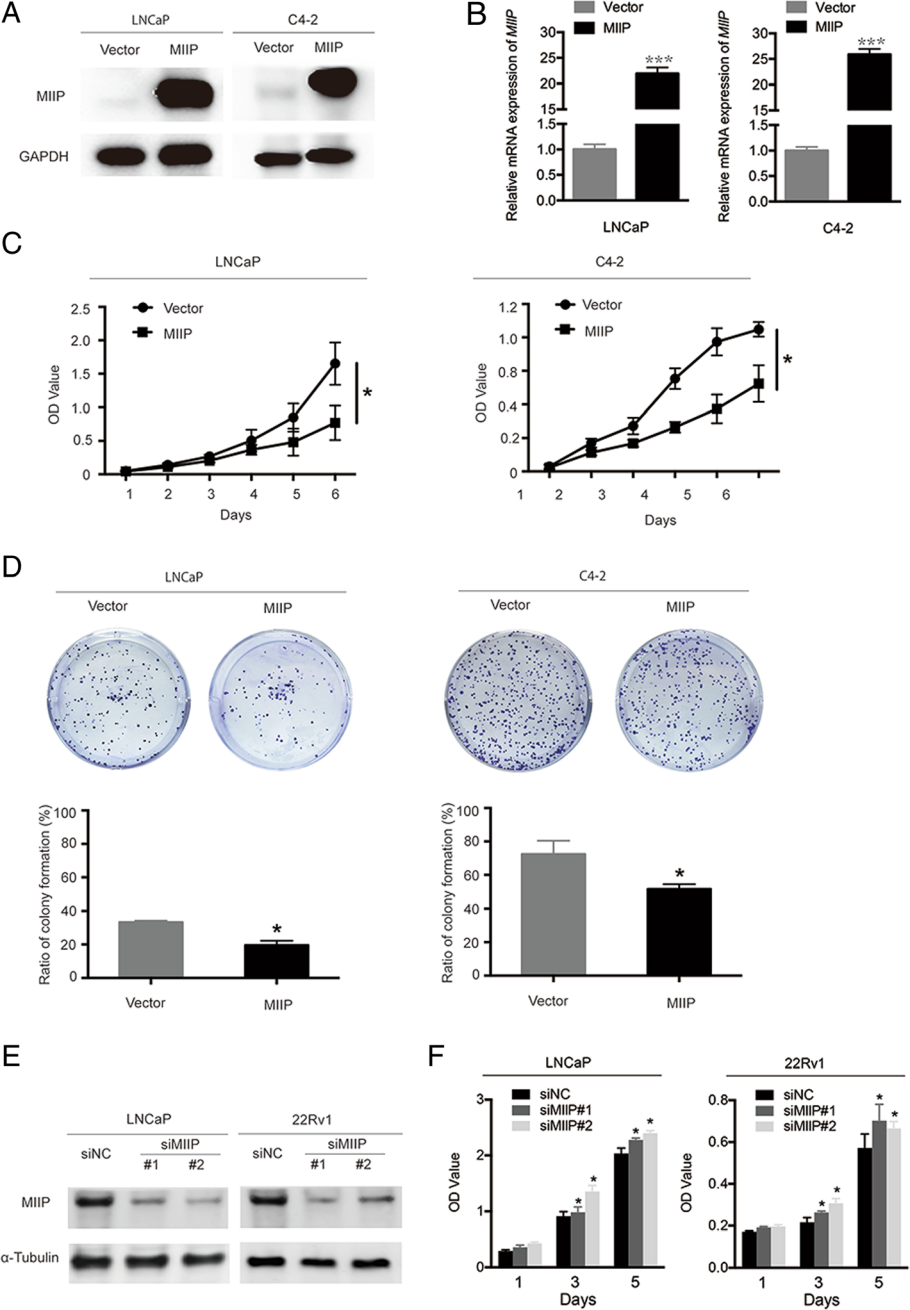
MIIP inhibits PCa cell proliferation. (**a**) Western blot analysis of MIIP protein expression in stable control and MIIP-overexpressing LNCaP and C4–2 cell lines. (**b**) Real-time RT-PCR analysis of *MIIP* mRNA level in stable cell lines. (**c**) The cell viability was determined by CCK8 assays at indicated time points. (**d**) Relative colony formation of stable cell lines. (**e**, **f**) LNCaP and 22Rv1 cells were transfected with siNC or two individual MIIP-targeting siRNAs. MIIP protein expression was examined by Western blot analysis (**e**) and the cell viability was determined by CCK8 assays (**f**). Data represent means ± SEM.^*^p < 0.05

To investigate the role of MIIP in PCa cells, we established MIIP-overexpressing stable cell lines (LNCaP-MIIP and C4–2-MIIP) by lentivirus infection. Stably overexpressed MIIP in these two cell lines were confirmed by quantitative RT-PCR and Western blot analysis (Fig. 1a and b). CCK8 assay and plate colony formation assays were performed to evaluate the proliferation ability of MIIP-overexpressing LNCaP and C4–2 cells. It was shown that MIIP overexpression significantly decreased proliferation ability of PCa cell LNCaP and C4–2 (Fig. 1c). In addition, the ratio of colony formation in LNCaP-MIIP and C4–2-MIIP were much lower than those in control PCa cell (LNCaP: 33.27 ± 1.03% vs 19.53 ± 2.73%,*p<0.05; C4–2:72.33 ± 8.06% vs 51.53 ± 2.91%,*p<0.05, Fig. 1d). These results demonstrated that MIIP inhibits PCa cell proliferation.

Further, we examined if knockdown of MIIP affects cell proliferation inversely. To this end, LNCaP and 22Rv1 cells with relatively high expression of endogenous MIIP (Additional file 2: Figure S1) were transfected with MIIP-specific small interfering RNAs (siMIIP#1 and siMIIP#2), and then cell proliferation was evaluated. It was shown that when MIIP was knocked down efficiently (Fig. 1e), LNCaP and 22Rv1 cell proliferation were significantly promoted (Fig. 1f). Taken together, MIIP inhibits PCa cell proliferation and its downregulation causes increased proliferation.

### MIIP is not involved in regulation of transcription activity and nuclear translocation of androgen receptor

**Fig. 2:**
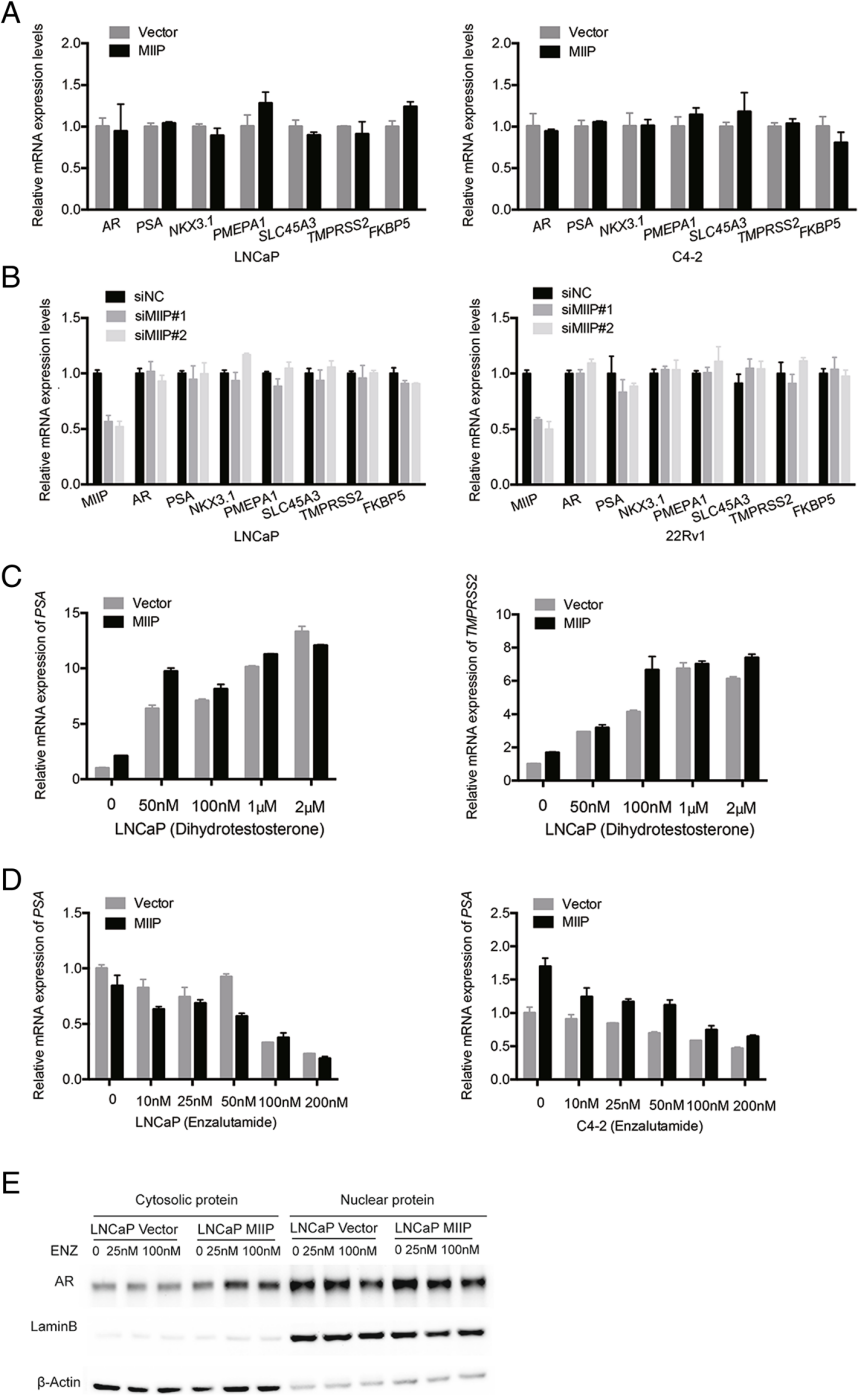
MIIP does not affect AR transcription activity and nuclear translocation. (**a**, **b**) Real-time RT-PCR analysis of *AR* and its target genes (*PSA,NKX3.1, PMEPA1, SLC45A3, TMPRSS2, FKBP5*) in stable control and MIIP-overexpressing LNCaP and C4–2 cells (A) and in LNCaP and 22Rv1 cells transfected with siNC or two individual MIIP-targeting siRNAs. (**c**) Real-time RT-PCR analysis of *PSA* and *TMPRSS2* in stable control and MIIP-overexpressing LNCaP cells treated with Dihydrotestosterone (0, 50 nM,100 nM, 1 μM and 2 μM). (**d**) Real-time RT-PCR analysis of *PSA* in stable control and MIIP-overexpressing LNCaP and C4–2 cells treated with Enzalutamide (0, 10, 25, 50,100 and 200 nM). (**e**) Western blot analysis of cytosolic and nuclear AR protein in stable control and MIIP-overexpressing LNCaP cells treated with Enzalutamide (0, 25, 100). Data were means ± SEM in **a**-**d**

AR signaling is the hallmark of PCa initiation and progression. Aberrant activation of AR signaling plays a critical role in the development of castration-resistant prostate cancer (CRPC) [5, 6]. To evaluate whether MIIP is involved in the regulation of AR signaling, we first examined the mRNA expression of *AR* and its target genes (*PSA,NKX3.1, PMEPA1, SLC45A3,TMPRSS2, FKBP5*) in LNCaP-MIIP and C4–2-MIIP by quantitative RT-PCR. The results showed that there were no differences of the mRNA expression level of *AR* and its target genes between MIIP-overexpressing PCa cells and control cells (Fig. 2a). We also checked the expression of AR and its target genes in LNCaP and 22Rv1 cells in which MIIP were knocked down by transient transfection with MIIP-targeting siRNAs. As shown in Fig. 2b, knockdown of MIIP had no effect on the transcription of AR and its target genes. To further determine whether MIIP is related to AR signal regulation, we pretreated LNCaP-MIIP and LNCaP-Vector with 50 nM-2 μM Dihydrotestosterone, an AR ligand capable of inducing AR activation, and then examined the expression of AR target gene *PSA* and* TMPRSS2*. The results showed that the mRNA expression level of *PSA* and *TMPRSS2* were simultaneously increased with the increase of Dihydrotestosterone concentration and there were no differences between LNCaP-MIIP and LNCaP-Vector (Fig. 2c). This suggested that MIIP does not affect ligand-induced AR activation. We further tested if MIIP affects AR regulation by Enzalutamide, a novel AR antagonist which inhibits AR nuclear translocation and chromatin binding [7]. Quantitative RT-PCR analysis suggested that the inhibition pattern of *PSA* transcript was similar between MIIP-overexpressing LNCaP or C4–2 and the corresponding control cells upon treated with Enzalutamide (10-200 nM) (Fig. 2d). Western blot analysis with cytosolic and nuclear protein showed there was no difference of AR nuclear translocation between LNCaP-MIIP and LNCaP-Vector cells when treated with Enzalutamide (25-100 nM) (Fig. 2e). Take together, these data suggested that MIIP is not involved in the regulation of transcriptional activity of AR and its nuclear translocation.

### MIIP inhibits AKT-mTOR signaling activation in PCa cells

**Fig. 3:**
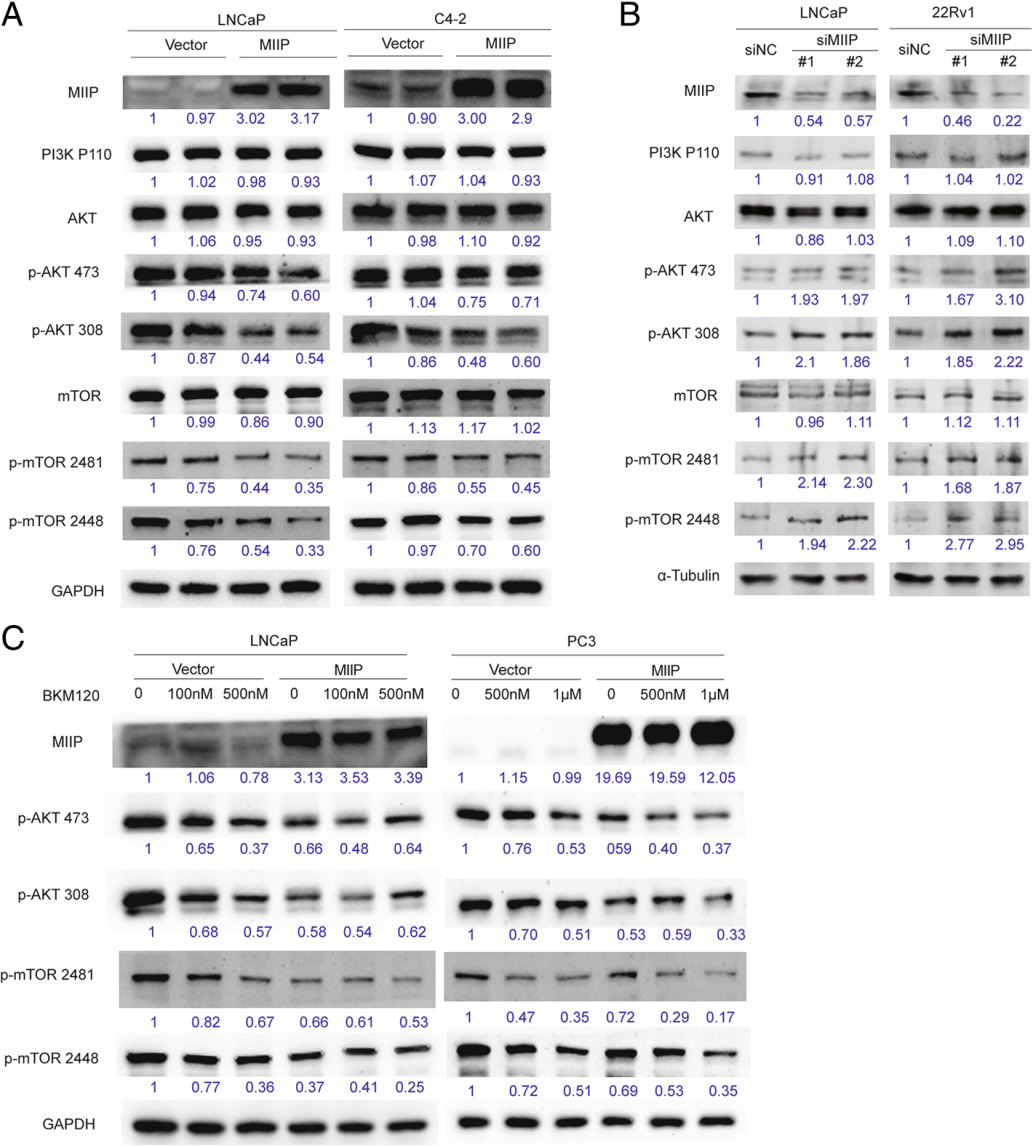
MIIP negatively regulates AKT-mTOR signaling. (**a**, **b**) Western blot analysis of key components of PI3K-AKT-mTOR signaling pathway [PI3K (p110), AKT, p-AKT (Ser473), p-AKT (Thr308), p-mTOR (Ser2481) and p-mTOR (Ser2448)] in stable control or MIIP-overexpressing LNCaP and C4–2 cells, and in LNCaP and 22Rv1 cells transfected with siNC or two individual MIIP-targeting siRNAs . (**c**) Western blot analysis of p-AKT (Ser473), p-AKT (Thr308), p-mTOR (Ser2481) and p-mTOR (Ser2448) in stable control and MIIP-overexpressing LNCaP and PC3 cells treated with BKM120 (0, 100 nM, 500 nM, or 1 μM). Each band’s intensity was quantified by Image J software, with the relative values (GAPDH or tubulin as internal control) of the left-most band being designated as 1 and the values of the others relative to it shown below

PI3K-AKT-mTOR pathway is another key signaling pathway that has been linked to both tumorigenesis and resistance to therapy in PCa [9]. Activation of the PI3K-AKT-mTOR pathway leads to enhanced PCa cell proliferation, survival and migration as well as castration-resistant progression [9]. To explore the relationship between MIIP and PI3K-AKT-mTOR signaling, we examined the expression of key components of PI3K-AKT-mTOR signaling pathway by Western blot analysis. It was shown that p-AKT (Ser473), p-AKT (Thr308), p-mTOR (Ser2481) and p-mTOR (Ser2448) were significantly down-regulated by MIIP overexpression in LNCaP and C4–2(Fig. 3a), but upregulated by MIIP knockdown in LNCaP and 22Rv1 cells (Fig. 3b), while there were no change in PI3K(p110), total AKT and mTOR (Fig. 3a and b). To further verify the effect of MIIP on AKT-mTOR signaling, we checked the signaling activation in both AR-positive LNCaP cells and AR-negative PC3 cells treated with or without PI3K inhibitor BKM120. As shown in Fig. 3c, BKM120 effectively decreased levels of p-AKT (Ser473), p-AKT (Thr308), p-mTOR (Ser2481) and p-mTOR (Ser2448) with a dose-dependent manner. Moreover, there was an additive effect on p-AKT and p-mTOR between MIIP-overexpression and BKM120 (100 nM), especially in LNCaP cells. Taken together, these data suggested that MIIP inhibits AKT-mTOR signaling downstream of PI3K and cooperates with PI3K inhibitor.

### MIIP attenuates AKT-mTOR axis by interacting with serine/threonine protein phosphatase 1α (PP1α)

**Fig. 4:**
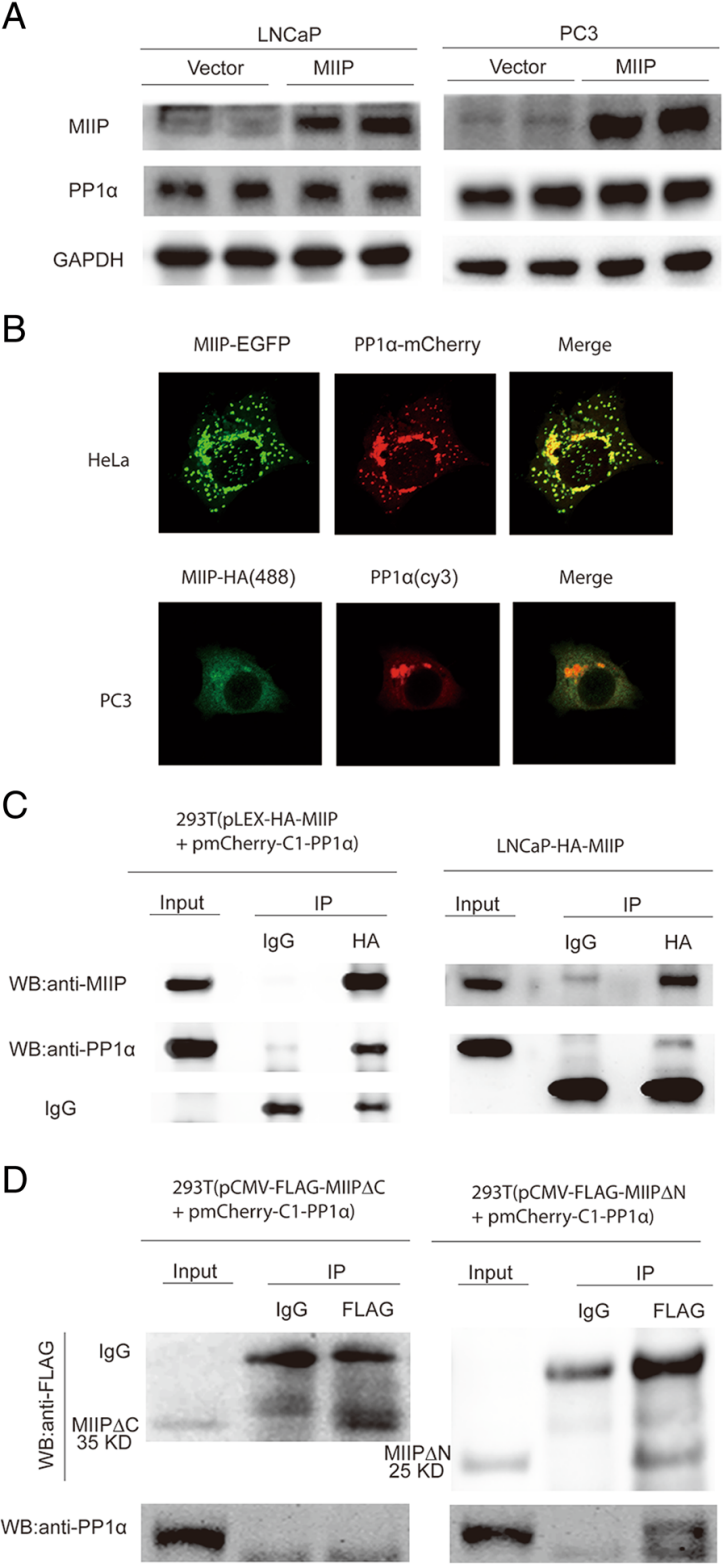
MIIP interacts with Serine/Theonine Protein Phosphatase PP1α via its C-terminal part. (**a**) Western blot analysis of PP1α in stable control and MIIP-overexpressing LNCaP and PC3 cells. (**b**) Co-localization analysis MIIP and PP1α in HeLa cells co-transfected with pEGFP-N3-MIIP and pmCherry-C1-PP1α and in MIIP-HA-LNCaP cells transfected with pmCherry-C1-PP1α. (**c**) Co-immunoprecipitation analysis of MIIP and PP1α in 293 T cells co-transfected with pLEX-HA-MIIP and pmCherry-C1-PP1α, and in LNCaP-HA-MIIP cells transfected with pmCherry-C1-PP1α, by immunoprecipitation with anti-HA and immunoblot with anti-PP1α. (**d**) 293 T cells were co-transfected with pmCherry-C1-PP1α and pFLAG-CMV4-MIIP∆C or pFLAG-CMV4-MIIP∆N, and co-immunoprecipitation analysis were performed by immunoprecipitation with anti-FLAG and immunoblot with anti-PP1α

The AKT-mTOR axis is activated by PI3K, and negatively regulated by several phosphatases such as PTEN [11]. We have revealed that MIIP has no influence on catalytic subunit of PI3K P110 (Fig. 3a and b). Moreover, since MIIP was able to negatively regulate AKT-mTOR signaling in both PTEN-deficient PCa cell LNCaP,C4–2 and PC3 and in PTEN-intact 22Rv1 cells (Fig. 3a and b), we concluded that PTEN is not a critical mediator in this process. On the other hand, AKT-mTOR signaling can also be controlled by serine/threonine protein phosphatase PP1 and PP2A [14] and a previous yeast two-hybrid assay using PP1α as bait identified MIIP as one of PP1α-interacting proteins [26]. Therefore, we asked whether MIIP inhibits AKT-mTOR signaling through interacting with PP1α and enhancing dephosphorylation of AKT by PP1α. For this purpose, we detected PP1α protein level and MIIP-PP1α interaction in MIIP-overexpressing cells. Western blot analysis showed that MIIP has no effect on PP1α expression in LNCaP and PC3 (Fig. 4a). Immunofluorescence analysis showed that MIIP and PP1α have extensive colocalization in cytoplasm in HeLa cells transfected with pEGFP-N3-MIIP together with pmCherry-C1-PP1α and in MIIP-overexpressing PC3 cells transfected with pmCherry-C1-PP1α (Fig. 4b), suggesting their potential interaction. Then we performed co-immunoprecipitation assay in MIIP and PP1α co-overexpressing 293 T cells and in MIIP-overexpressing LNCaP cells and found there was a strong interaction between MIIP and PP1α (Fig. 4c). To determine which part of MIIP is responsible for such interaction, we made FLAG-tagged MIIP∆C and MIIP∆N constructs containing N-terminal 1–219 residues and C-terminal 220–388 residues of MIIP respectively. With these truncations, we further performed co-immunoprecipitation assay and demonstrated that MIIP∆N, but not MIIP∆C, binds with PP1α (Fig. 4d).

**Fig. 5:**
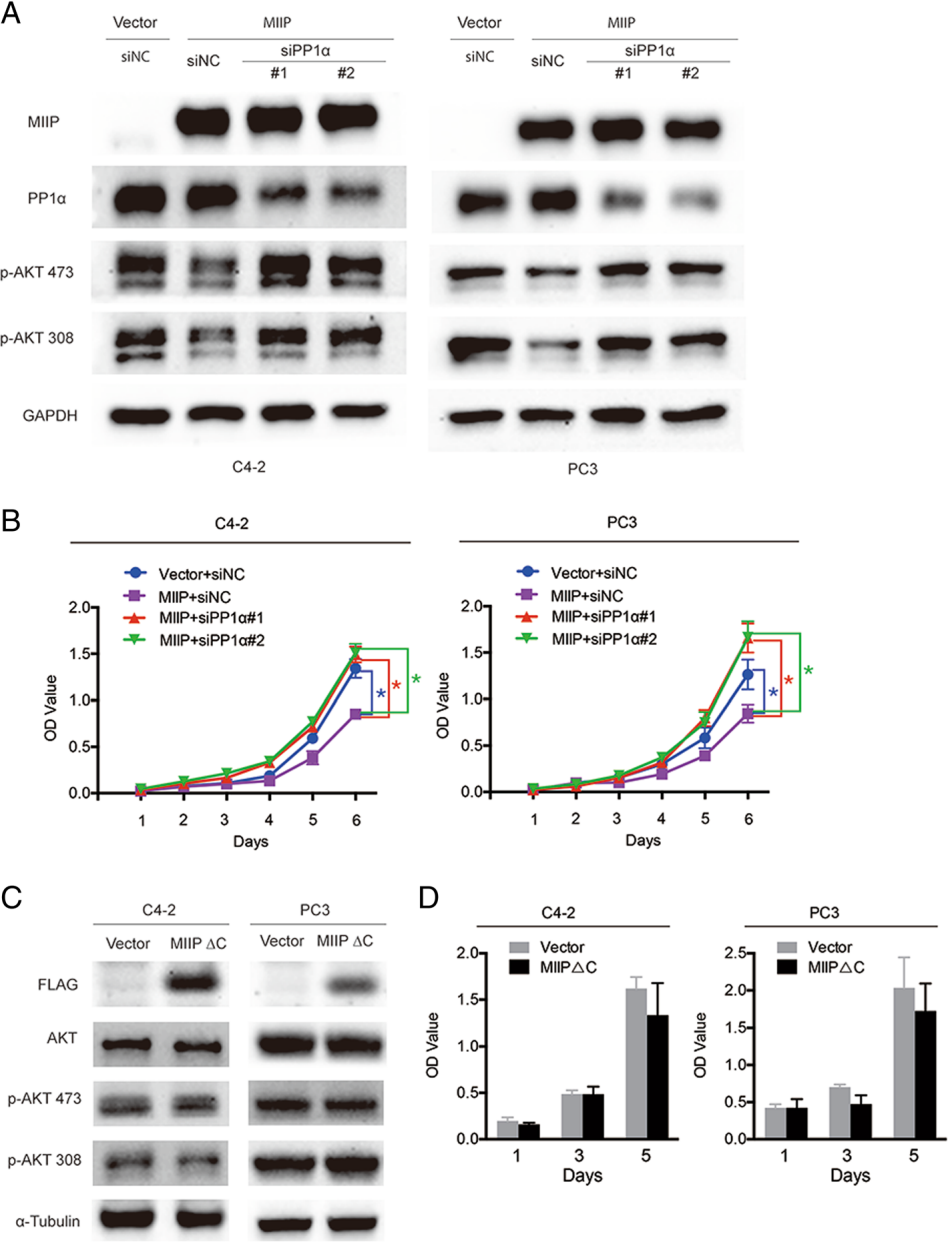
MIIP’s effect on AKT phoshphorylation and cell proliferation relies on interaction with PP1α. (**a**, **b**) C4–2-MIIP and PC3-MIIP cells were transfected with siNC, siPP1α#1 and siPP1α#2. Western blot analysis of p-AKT (Ser473) and p-AKT (Thr308) (**a**) and CCK8 assays analysis of cell viability (**b**) were performed. (**c**, **d**) C4–2 and PC3 cells were transiently transfected with pFLAG-CMV4-MIIP∆C or empty vector and 48 h later, subjected to Western blot and CCK8 assays. Data were means ± SEM. ^*^p < 0.05

To determine whether MIIP attenuates AKT-mTOR axis via PP1α, we used PP1α-specific small interfering RNAs (siPP1α#1 and siPP1α#2) to silence the endogenous PP1α in C4–2 and PC3 and then examined MIIP’s effect on AKT phosphorylation. Western blot analysis showed that MIIP-caused decrease in p-AKT could be reversed when PP1α was silenced (Fig. 5a). Similarly, PP1α silence abrogated MIIP’s function of inhibiting PCa cell proliferation (Fig. 5b). Since MIIP∆C was not able to interact with PP1α (Fig. 4d), we then asked if MIIP∆C lost the function of MIIP. To this end, C4–2 and PC3 cells were transiently transfected with pFLAG-CMV4-MIIP∆C or empty vector and then subjected to Western blot and CCK8 analysis. As shown in Fig. 5c and d, MIIP∆C had no effect on either AKT phosphorylation or cell proliferation compared with vector control. Collectively, these data clearly indicated that MIIP inhibits PCa cell proliferation by interacting with PP1α though its C-terminal part, facilitating PP1α-mediated AKT dephosphorylation and thus leading to attenuation of AKT-mTOR axis.

### MIIP inhibits tumor growth in xenograft mouse model of PCa

**Fig. 6:**
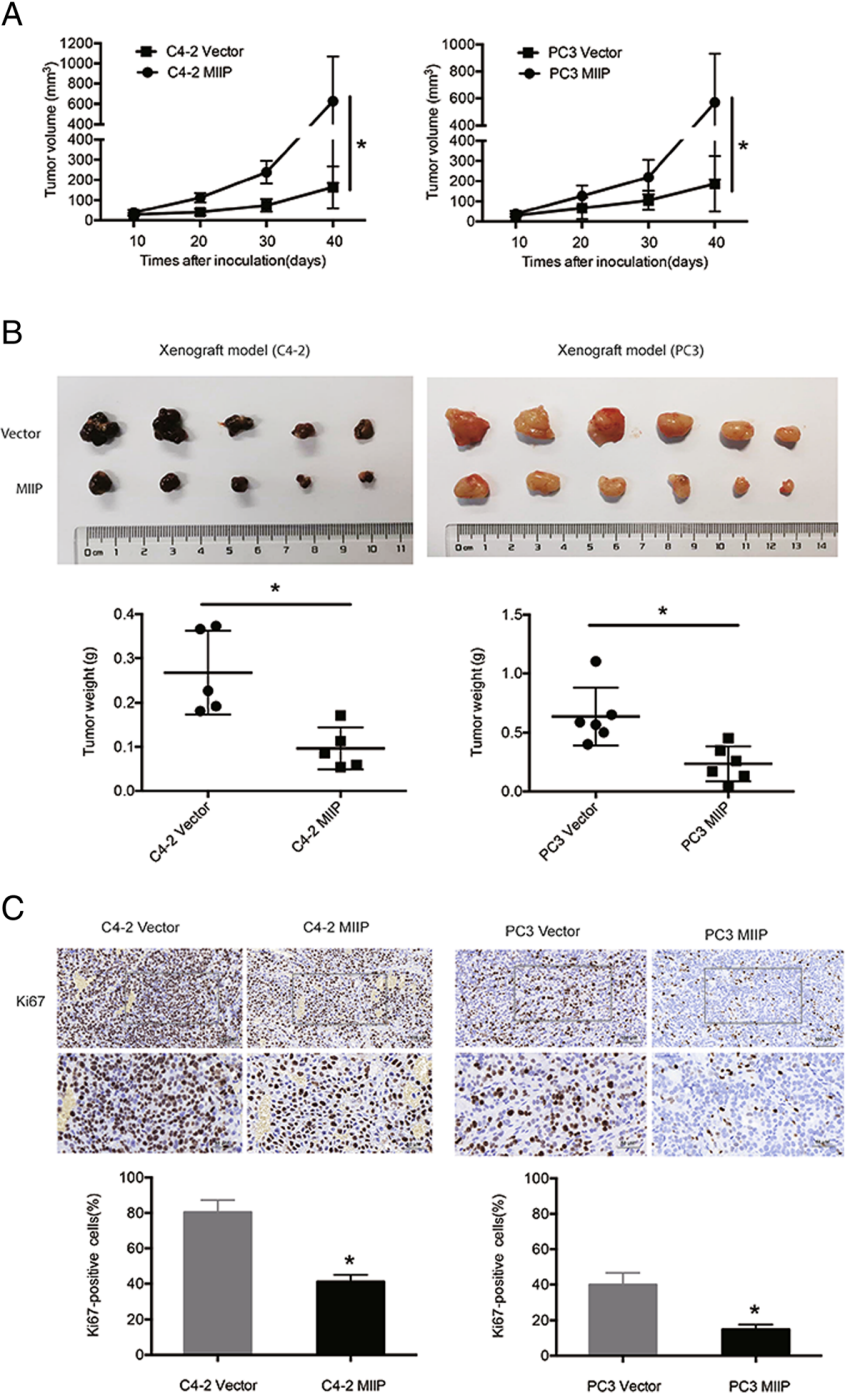
MIIP inhibits tumor growth in xenograft mice models of PCa. (**a**) The tumor growth curve of xenograft derived from C4–2-Vector, C4–2-MIIP, PC3-Vector and PC3-MIIP. (**b**) Images and tumor weight were obtained at 40 day after transplantation. (**c**) The tumor derived from the stable PCa cells were subjected to immunostaining for Ki67. Data were means ± SEM. ^*^p < 0.05

To further confirm the tumor-suppressive function of MIIP in PCa, we examined the effects of MIIP over-expression on in vivo tumor growth in xenograft mouse model. After inoculation with PCa cells, tumor volume was monitored ever 10 days. As shown in Fig. 6a, the nude mice injected with MIIP-overexpressing C4–2 and PC3 developed tumors more slowly than those injected with their corresponding control cells. As a result, the final tumor sizes of nude mice injected with MIIP-overexpressing C4–2 and PC3 cells were much smaller than those injected with C4–2 and PC3 vector control cells (C4–2: 0.27 ± 0.09 vs 0.09 ± 0.05 g, *p<0.05; PC3: 0.63 ± 0.24 vs 0.23 ± 0.15, *p<0.05; Fig. 6b). Ki67 staining analysis also confirmed that MIIP overexpression inhibits PCa cells proliferation (Fig. 6c). Take together, in vivo study further emphasized the tumor suppressive role of MIIP in PCa.

### MIIP expression is downregulated in PCa and negatively correlated with clinical PCa progression

**Fig. 7:**
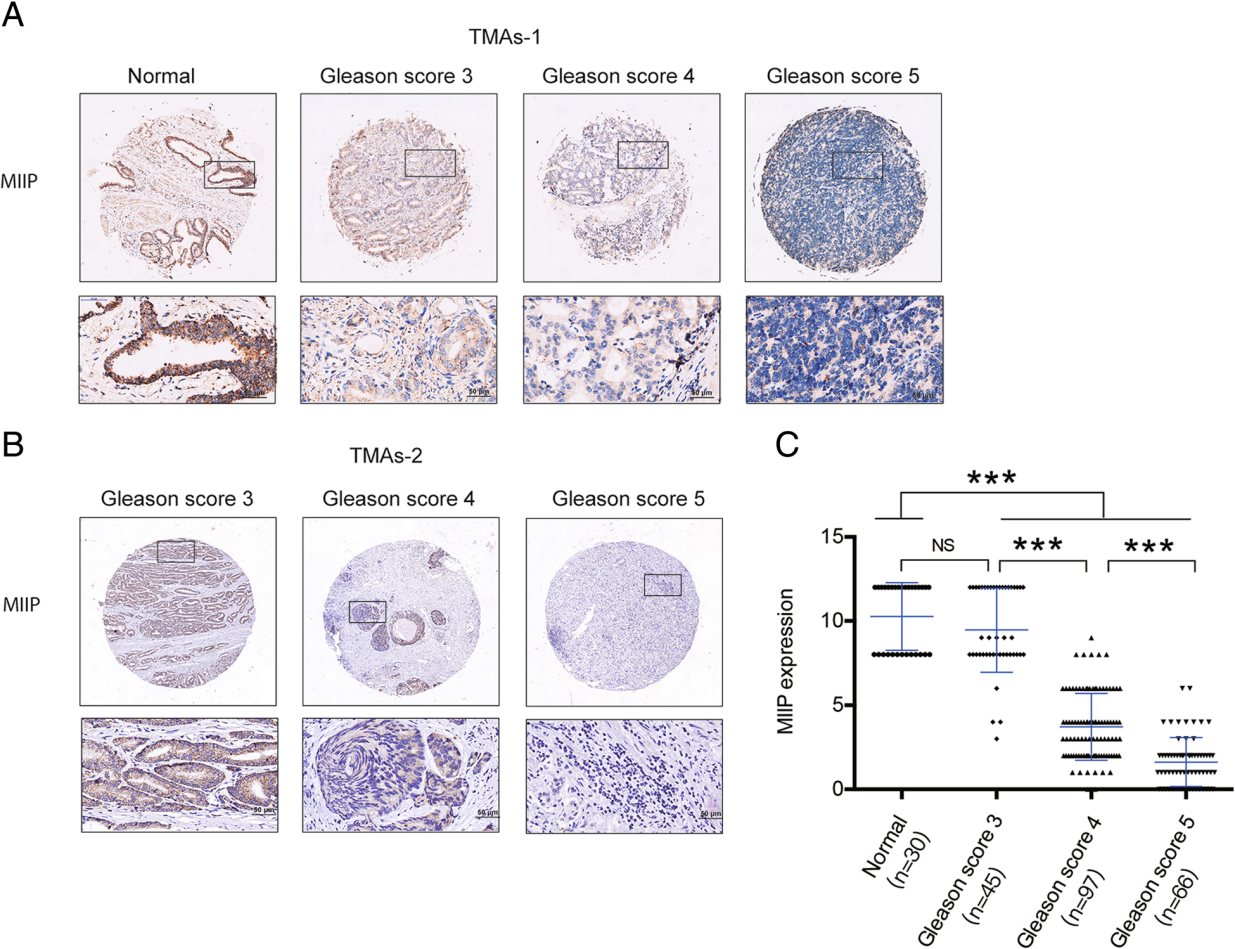
MIIP expression is downregulated in PCa and negatively correlated with PCa progression. MIIP expression was evaluated by immunohistochemical staining on two independent TMAs. Representative images (**a**, **b**) and relative expression level of MIIP (**c**) in normal and adjacent normal prostate tissues, and PCa tissues of Gleason score 3, 4 and 5 grades. Scale bar, 50 μm. Data were means ± SEM. ^***^p < 0.001

Finally, to explore the clinical relevance of MIIP in PCa, we analyzed the expression of MIIP in two independent tissue microarrays (TMAs), which contain normal and adjacent normal prostate tissue, and PCa tissues of different Gleason score, by immunohistochemistry (IHC). The staining result showed that the expression of MIIP decreased significantly along with the increase of PCa Gleason score (Fig. 7), albeit there was no significant difference between (adjacent) normal prostate tissue and Gleason score 3. These results clearly indicated that MIIP expression is downregulated in PCa and negatively correlated with PCa progression. Taken these cytological and xenograft model studies together, MIIP plays a strong tumor-suppressive function and its downregulation may contribute to PCa progression.

## Discussion

MIIP is a newly identified tumor suppressor in many different types of cancer, including glioma, lung cancer, colon cancer, endometrial carcinoma, pancreatic cancer and others [18–20, 22, 24, 25], yet its role in prostate cancer has not been reported and its biological function and the relevant mechanism is far from clarified. Here, we show that forced expression of MIIP inhibits the growth of both AR- positive and negative prostate cancer cell lines as well as the corresponding xenograft, while knockdown of MIIP does the opposite. In mechanism, MIIP has no effect on AR signaling but attenuates AKT-mTOR axis by interacting with Serine/Threonine Protein phosphatase PP1α through its C-terminal part and facilitating PP1α-dependent dephosphorylation of AKT. Moreover, MIIP expression level is downregulated in PCa and negatively correlated with the Gleason score of PCa. These data illustrate the novel mechanism through which MIIP exerts its inhibitory role in PCa.

As a migration and invasion inhibitory protein and a metastasis suppressor, MIIP was reported to bind to and antagonize the function of diverse effectors, including IGFBP-2 [20], HDAC6 [23] and PAK1 [18], depending on different molecular context in different cancer types. Besides, growing evidences have shown that MIIP also inhibits cell proliferation [19, 24, 25]. MIIP is able to interact with Cdc20 and suppress the activity of APC/C^Cdc20^, thus blocking the degradation of two mitotic check proteins, cyclin B1 and securing, leading to impaired mitotic transition in glioma and colon cancer model [19, 24]. Moreover, MIIP can accelerate EGFR degradation and inhibit downstream Ras/MEK/ERK signal pathway, resulting in inhibition of cell proliferation of non-small cell lung cancer [19]. Here we for the first time demonstrated that MIIP inhibits prostate cancer cell proliferation by interacting with PP1α and facilitating PP1α-dependent dephosphorylation of AKT and attenuation of downstream AKT-mTOR signaling pathway.

PP1 constitutes a major class of Ser/Thr protein phosphatase and is highly conserved among eukaryotes [27]. There are three PP1 catalytic subunits, i.e., PP1α, PP1β, and PP1γ, which are encoded by three different genes [28–30]. By catalyzing dephosphorylation of its substrates, PP1 regulates a variety of signaling pathway and cellular processes, particularly those related with protein (de) phosphorylation [31]. Therefore, the activity of PP1 itself is under strict, temporal and spatial regulation. More than 200 proteins have been identified to interact with PP1 [32]. Using yeast two-hybrid screening, a previous study revealed MIIP as one of PP1α-interacting proteins [26], suggesting it may function as regulator, substrate specifier or substrates itself for PP1α [32]. Our current study not only confirmed MIIP-PP1α interaction using co-immunoprecipitation assay and co-localization assay, but also revealed that C-terminal rather than N-terminal of MIIP accounts for such an interaction (Fig. 4). Moreover, we showed that MIIP facilitated PP1α-mediated dephosphorylation of AKT (Fig. 3), but MIIP∆C, which is incapable of interacting with PP1α, lost such a regulatory role for AKT phosphorylation and cell proliferation (Fig. 5). Notably, Li et al. demonstrated that the activity PP1α for AKT is limited to T308 and has no effect on S473 [14], but we showed here that PP1α is required for MIIP to decrease the phosphorylation level at both T308 and S473 because PP1α silence almost completely reversed the inhibitory effect of MIIP on these two phosphorylation sites (Fig. 5). Such discrepancy probably results from different experimental system and cellular context, whereby Li et al. examined dephosphorylation of p-AKT 308 and p-AKT 473 in cav-1-expressing, LY294002-treated LNCaP cell lysate in vitro using purified PP1 enzyme, while we examined p-AKT 308 and p-AKT 473 levels in MIIP-overexpressing, PP1α-silenced C4–2 and PC3 cells. Consistent with our conclusion, Xu et al. showed that PP1 associates with and directly dephosphorylates AKT at S473 using purified PP1 and AKT in an in vitro phosphatase assay [15]. Moreover, PP1-dependent dephosphorylation of AKT is vulnerable to regulation by oncoproteins or anti-cancer agents [15, 16]. For example, ErbB2 inhibits PP1-dependent dephosphorylation of AKT in breast cancer cells whereas ErbB inhibitor and Hsp90 inhibitor promote such an event [15]. In addition, HDAC inhibitors could facilitate dephosphorylation of AKT by disrupting HDAC-PP1 complexes while enhancing PP1-AKT association [16]. Here we further added to the landscape that PP1-mediated dephosphorylation AKT is subjected to regulation by tumor suppressor. More interestingly, the most recent study reported that MIIP interacts with PP1 and serves as its substrate of dephosphorylation at S303, which undergoes phosphorylation by PKCε upon EGF-treatment in colon cancer [33]. Therefore, it deserves further investigation as whether MIIP serves as a PP1 regulator or a substrate in different cancer type, and whether there exists a reciprocal regulatory relationship between them.

Importantly, our current study revealed that, by facilitating PP1α-mediated dephosphorylation of AKT, MIIP inhibits the downstream AKT-mTOR pathway regardless of AR positive/negative status and androgen-sensitive/castration-resistant status (Fig. 3). Moreover, MIIP seems not to affect catalytic subunit of PI3K, P110, which functions as an important upstream regulator of AKT, but cooperates with P110 inhibitor to further restrict AKT-mTOR signaling (Fig. 3). Additionally, we found that MIIP has no effect on AR signaling, evinced by the result that there is no difference either in transcription of AR target genes or in AR translocation, even upon treatment with AR ligand or inhibitor (Fig. 2). It has been known that PI3K-AKT-mTOR and AR signaling interplay with each other to promote CRPC, and inhibition of one pathway always drive reciprocal activation of the other pathway [9]. As such, MIIP-mediated inhibition of AKT-mTOR in combination with AR inhibition could serve as a promising therapeutic modality for CRPC.

## Conclusion

In summary, we for the first time demonstrated here that MIIP is a novel suppressor of prostate cancer and the higher the Gleason score, the lower its expression. We also revealed the underlying mechanism through which MIIP exerts its tumor-suppressive role by interacting with PP1α and facilitating dephosphorylation of AKT, thereby leading to attenuation of AKT-mTOR axis. Combination of such suppressive role of MIIP with the inhibitor of AR signaling may provide new therapeutic clue for CRPC treatment.

## Additional files

Additional file 1: **Table S1.** Primers used for vector construct. **Table S2.** Primers for qRT-PCR analysis. (DOCX 15 kb)

Additional file 2: **Figure S1.** Endogenous MIIP expression in different prostate cancer cell lines. Cells were lysed and equal amount of cell lysates were subjected to Western blot analysis with anti-MIIP and anti-GAPDH respectively. (TIF 63 kb)

## Abbreviations

AR: Androgen receptor
CRPC: Castration resistant prostate cancer
MIIP: Migration and invasion inhibitor protein
mTOR: Mammalian target of rapamycin
PCa: Prostate cancer
PI3K: Phosphatidylinositol 3-kinase (PI3K)
PP1: Protein phosphatase 1
