# Correction to: MIIP inhibits the growth of prostate cancer via interaction with PP1α and negative modulation of AKT signaling

**DOI:** 10.1186/s12964-019-0441-4

**Published:** 2019-10-21

**Authors:** Guang Yan, Yi Ru, Fengqi Yan, Xin Xiong, Wei Hu, Tao Pan, Jianming Sun, Chi Zhang, Qinhao Wang, Xia Li

**Affiliations:** 1 State Key Laboratory of Cancer Biology, Department of Biochemistry and Molecular Biology, The Fourth Military Medical University, Xi’an, 710032 Shaanxi China; 2 Andrology Department, Shanghai Seventh People’s Hospital, Shanghai, 200137 China; 3 Department of Urology, Tangdu Hospital, TheFourth Military Medical University, Xi’an, 710038 Shaanxi China; 4 Rehabilitation Department, Gongli Hospital of Shanghai Pudong New Area, Shanghai, 200137 China

**Correction to: Cell Commun Signal**

**https://doi.org/10.1186/s12964-019-0355-1**

Following publication of the original article [1], the authors reported that the given name of Qinhao Wang was incorrectly published as Qinghao Wang. The original article has been corrected.
