# Supplementary material for: MIIP inhibits the growth of prostate cancer via interaction with PP1α and negative modulation of AKT signaling
Source: Cell Commun Signal. 2019 May 15;17:44. doi: 10.1186/s12964-019-0355-1 (PMC6521544; doi:10.1186/s12964-019-0355-1)
Supplement: Supplementary file 1 — Table S1. Primers used for vector construct. Table S2. Primers for qRT-PCR analysis. (DOCX 15 kb) [file 12964_2019_355_MOESM1_ESM.docx]

Guang Yan^1, 2#^, Yi Ru^1#^, Fengqi Yan^1, 3#^, Xin Xiong^1^, Wei Hu^3^, Tao Pan^1^, Jianming Sun^2^, Chi Zhang^4^, Qinghao Wang^1*^, Xia Li^1*^

^1^State Key Laboratory of Cancer Biology, Department of Biochemistry and Molecular Biology, The Fourth Military Medical University, Xi'an, Shaanxi 710032, China

^2^Andrology Department, Shanghai Seventh People's Hospital, Shanghai 200137, China

^3^ Department of Urology, Tangdu hospital, The Fourth Military Medical University, Xi'an, Shaanxi 710038, China

^4^ Rehabilitation Department , Gongli Hospital of Shanghai Pudong New Area, Shanghai 200137, China.

*Correspondence: Xia Li and Qinhao Wang, e-mail: [lixia@fmmu.edu.cn](mailto:lixia@fmmu.edu.cn) and [rayn1222@sina.com](mailto:rayn1222@sina.com)

#These authors contributed equally to this work.

Table S1. Primers used for vector construct.

| Construct name | Forward Primer (5'–3') | Reverse Primer (5'–3') |
| --- | --- | --- |
| pLEX-HA-MIIP | CGGACTAGTATGGTGGAGGCTGAGGAAC | CCGCTCGAGTCCTCCCTCTCTGGAACTCA |
| pFLAG-CMV4-MIIP-N | ggcgaattcAatggtggaggctgaggaactg | ggcgatatctcaTtcaggatggctgcag |
| pFLAG-CMV4-MIIP-C | ggcgaattcAatgGAaccccagttgccaggcct | ggcgatatctcagggcttctgccgtgg |
| pEGFP-N3-MIIP | ggcgctagcatggactacaaagacgatgacgacaag | AAGCTTggggcttctgccgtgggac |
| pmCherry-C1-PP1α | CCGGAATTCATGTCCGACAGCGAGAAGCTCAACCT | CGCGGATCCCTATTTCTTGGCTTTGGCGGA |

Table S2. Primers for qRT-PCR analysis.

| Gene name | Forward Primer (5'–3') | Reverse Primer (5'–3') |
| --- | --- | --- |
| *MIIP* | ATACCTGGGCTATGACTGGATT | AGTACACGCATTCATGGTCTTC |
| *PSA* | CACCTGCTCGGGTGATTCTG | CCACTTCCGGTAATGCACCA |
| *TMPRSS2* | GTCCCCACTGTCTACGAGGT | CAGACGACGGGGTTGGAAG |
| *AR* | CTCCGCTGACCTTAAAGACATC | TGCCCCCTAAGTAATTGTCCTT |
| *NKX3.1* | ACTTGGGGTCTTATCTGTTGGA | CTCGATCACCTGAGTGTGGG |
| *PMEPA1* | TGTCAGGCAACGGAATCCC | CAGGTACGGATAGGTGGGC |
| *SLC45A3* | CCTTCACGCTGTTTTACACGG | CGCCTTCATCATAGTGTCTCC |
| *FKBP5* | CATCAAGGCATGGGACATTGG | TCGAGGGAATTTTAGGGAGACT |
| *GAPDH* | CCATCTTCCAGGAGCGAGATC | GCCTTCTCCATGGTGGTGAA |

**Fig. S1. Endogenous MIIP expression in different prostate cancer cell lines.** Cells were lysed and equal amount of cell lysates were subjected to Western blot analysis with anti-MIIP and anti-GAPDH respectively.
