# Supplementary figures and images for: MIIP inhibits the growth of prostate cancer via interaction with PP1α and negative modulation of AKT signaling
Source: Cell Commun Signal. 2019 May 15;17:44. doi: 10.1186/s12964-019-0355-1 (PMC6521544; doi:10.1186/s12964-019-0355-1)

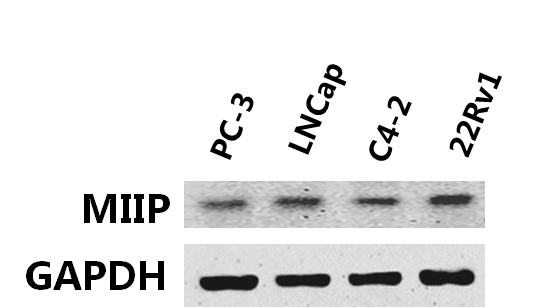

Supplement: Supplementary file 2 — Figure S1. Endogenous MIIP expression in different prostate cancer cell lines. Cells were lysed and equal amount of cell lysates were subjected to Western blot analysis with anti-MIIP and anti-GAPDH respectively. (TIF 63 kb) [file 12964_2019_355_MOESM2_ESM.tif]
